# Contemporary clinical updates on the prevention of future cardiovascular disease in women who experience adverse pregnancy outcomes

**DOI:** 10.1002/clc.23374

**Published:** 2020-04-17

**Authors:** Ki Park, Margo B. Minissian, Janet Wei, George R. Saade, Graeme N. Smith

**Affiliations:** ^1^ Department of Medicine, Division of Cardiology University of Florida Gainesville Florida USA; ^2^ Barbra Streisand Women's Heart Center Smidt Heart Institute, Cedars‐Sinai Medical Center Los Angeles California USA; ^3^ Department of Obstetrics and Gynecology University of Texas Medical Branch Galveston Texas USA; ^4^ Department of Biomedical and Molecular Sciences Queen's University Kingston Ontario Canada

**Keywords:** adverse pregnancy outcomes, cardiovascular risk, women

## Abstract

Adverse pregnancy outcomes including hypertensive disorders of pregnancy and gestational diabetes are significant causes of maternal mortality. There is substantial evidence of an association between adverse events during pregnancy and long‐term maternal cardiovascular risk. It is therefore important to understand the role of risk modification prior to, during, and after pregnancy to reduce adverse outcomes. These efforts include risk assessment, routine screening for cardiovascular risk factors, and potential pharmacotherapeutic risk reduction. In this manuscript, we aim to highlight the current evidence in the areas of cardiovascular risk assessment and risk modification, and the role for potential risk reduction therapies before, during, and after pregnancy.

## 1 INTRODUCTION

**FIGURE 1:**
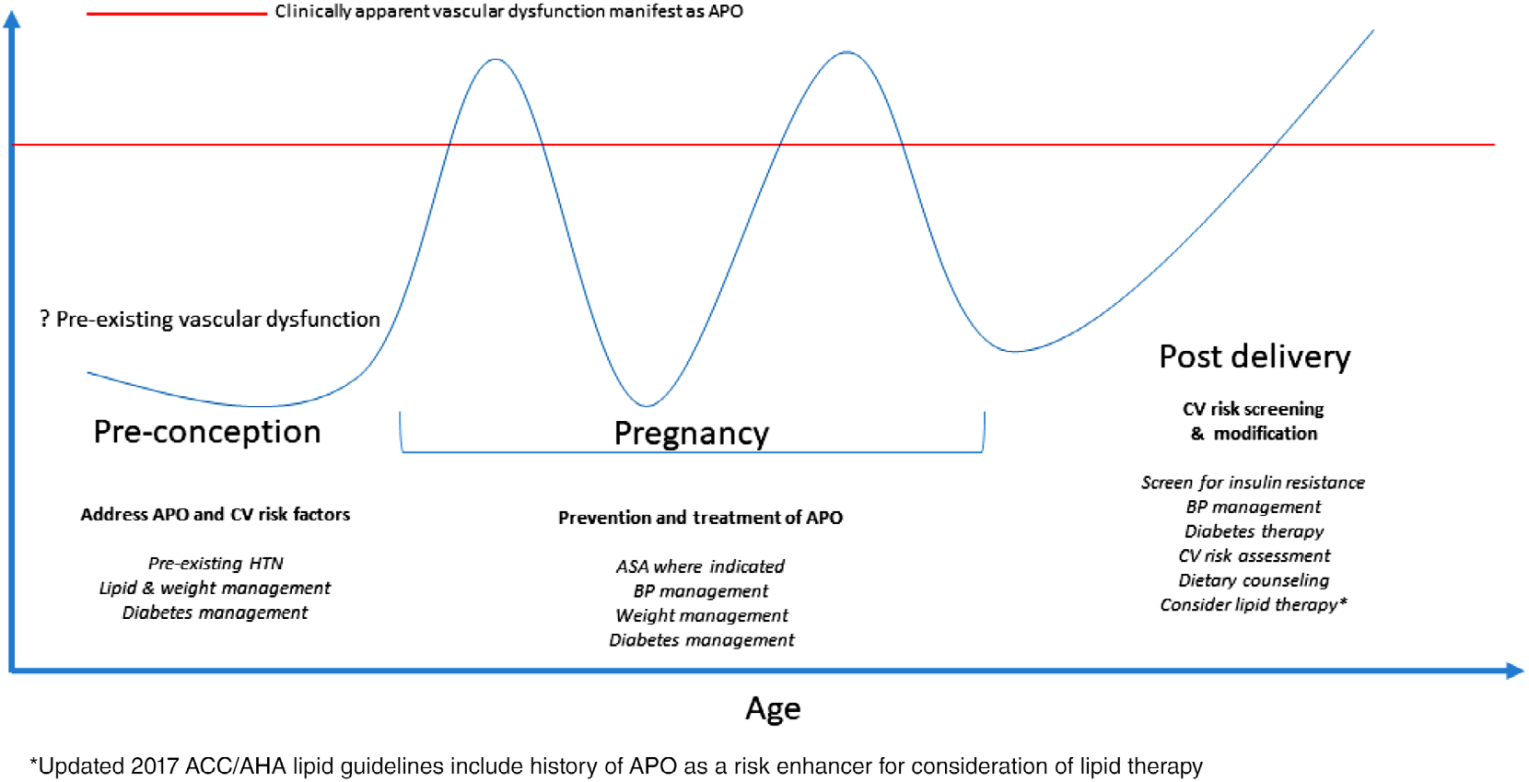
Life course of adverse pregnancy outcomes (APO) and cardiovascular (CV) risk assessment, treatment, and modification. Building on the work from Sattar and Greer,^14^ this figure details recommendations for pre‐, during, and postpregnancy to further reduce future maternal CV disease risk

Cardiovascular disease (CVD) remains the leading cause of death for women in the United States, responsible for one in three deaths.^1^ As maternal age increases and more women with congenital conditions enter child‐bearing years, the impact of cardiovascular health on maternal and neonatal outcomes has become more apparent as evident from data from registries such as the Registry Of Pregnancy And Cardiac disease and associated guidelines in both Europe and the United States.^2^, ^3^ In turn, women with adverse pregnancy outcomes (APOs) are also at increased risk of CVD, and APOs are now recognized as CVD risk‐identifying conditions that should initiate clinician‐patient risk discussion.^4^, ^5^ These APOs (ie, hypertensive disorders of pregnancy [HDPs], gestational diabetes, preterm birth at <37 weeks of gestation, and intrauterine growth restriction) are associated with a 2‐fold increased future CVD risk.^6^ Additional recent data suggests a broader spectrum of APOs including placental abruption and still birth may also be associated with CVD.^7^ Presence of APOs in multiple pregnancies increases the risk even more than if only one pregnancy is affected.^8^ Women with APOs are at risk for premature CVD events such as heart failure, ischemic heart disease, stroke, atrial arrhythmias, and cardiovascular death,^9^, ^10^ with women experiencing their first CVD event by a mean age of 38 years.^11^ Despite associations between APOs and future CVD, it remains unclear whether the cardiovascular changes associated with APOs causally lead to future CVD or if APOs are manifestations of underlying increased CVD risk. APOs are also associated with CVD risk factors, including a 4‐fold increase in hypertension,^12^ 7‐fold increase in diabetes,^13^ and a 5‐ to 12‐fold increased risk of renal disease.^9^ Given these associations, there is an urgent need to develop risk stratification tools, optimal screening strategies, and preventive interventions for women after a single or recurrent APO to deter future CVD. Such strategies to reduce APO and future CVD risk are discussed in the following sections and highlighted in the Figure 1.^14^

## 2 ROLE OF APOs IN CVD RISK PREDICTION

Standard CVD risk stratification models, which include consideration of sex, generally underestimate overall CVD risk in women, particularly young women.^15^ Although the recent 2018 American College of Cardiology (ACC)/American Heart Association (AHA) cholesterol guideline acknowledge APOs as CVD risk enhancers,^5^ none of the current risk calculators incorporates pregnancy history. Two recent studies were conducted in Scandinavian countries where robust birth registries provide opportunities for study. Markovitz et al^16^ utilized data from the HUNT study, which is an ongoing population‐based Norwegian cohort study. Investigators used the NORRISK 2 model to assess 10‐year risk of CVD (nonfatal myocardial infarction, fatal coronary heart disease, and nonfatal or fatal stroke). Among 18 000 women without prior history of CVD, 5% had a CVD event at 10‐year follow‐up, and 39% of all women studied experienced at least one APO (defined as preterm birth, HDP, and small for gestational age). Overall, women with prior APO history had a higher prevalence of traditional CVD risk factors associated with increased CVD risk. When adjusted for traditional CVD risk factors, only preeclampsia out of all APOs was associated with increased CVD risk. When including APO into risk modeling, 0.4% of women without events were reclassified into lower risk categories and 2% of women with events were correctly reclassified into higher risk categories. Therefore, adding APO led to only minimal improvement in risk prediction. A similar Swedish study from Timpka et al^17^ analyzed HDP and low birth weight of offspring and found similar results, although low birth weight was included (not included in the HUNT study) and age of subjects was limited to age ≥50 years. Compared with traditional risk factors, a history of HDP or delivery of a low birth weight infant did not significantly improve 10‐year CVD risk prediction in Scandinavian women age 50 years or older.

Investigators recently evaluated the clinical utility of incorporating HDP history into CVD risk prediction in a US population. Stuart et al studied >65 000 women age ≥40 years who did not have CVD risk factors or CVD at baseline.^18^ Diagnoses of CVD risk factors and CVD outcomes including myocardial infarction, fatal coronary heart disease, or stroke were collected over 25 to 32 years. HDP and parity were added to the atherosclerotic cardiovascular disease (ASCVD) Pooled Cohort Equations model. Among this cohort, 82% of women were parous and 10% had a history of HDP. HDP and parity were associated with elevated 10‐year ASCVD risk independent of established CVD risk factors in the overall population and in women ages 40 to 49 years, but not in older women age 50 to 59 years. However, C index and net reclassification index across risk group or age‐stratification did not differ. The model with HDP and parity correctly reclassified 0.6% of previously low‐risk women who developed CVD into a higher risk group but incorrectly reclassified 8.3% of previously intermediate‐risk women as low risk. Along similar lines, an additional study by Dam et al published in 2018 compared CVD risk prediction in women with and without a history of HDP among three different risk models including the Framingham Risk Score, the Pooled Cohort Equations model, and the Systematic Coronary Risk Evaluation (SCORE) model.^19^ In studying nearly 30 000 women, the authors found the SCORE model was the most predictive of CVD risk in women with HDP but overall none of the models performed any better in women with history of HDP vs women without history of HDP.

Although these studies did not demonstrate a definitive benefit to adding APO to risk models, there are several points to note. The lack of improvement in risk assessment seen in these recent studies may be due to underlying association between APOs and traditional risk factors such as elevated blood pressure and BMI which may lessen the relative association with the APO and CVD risk itself. However, there is still great utility in identifying women with APOs as CVD risk factors. Young women with APOs have elevated 10‐year CVD risk independent of established CVD risk factors, and would otherwise not undergo CVD risk factor screening or modification based on traditional risk scores. This suggests the importance of recognizing APOs as risk identifiers even if the APOs themselves do not improve the risk models. Additionally, there are limitations to the risk models. APO elements incorporated into these risk models varied from study to study. Additionally, although CVD endpoints varied between studies, none of them included heart failure as an outcome despite APOs, most notably HDP, being associated with significant risk of heart failure. Overall, the populations studied were low risk, and whether such analyses in higher risk populations such as African American women would lead to improved risk prediction is unknown. Additionally, these studies focused primarily focused on older women such as the study by Stuart et al which excluded women <40 years of age as the ASCVD Pooled Cohorts Equation risk model was not validated in this younger age group. However, these women are those who are most at risk from a lifetime risk perspective and thus the most likely to benefit from aggressive risk modification.^20^ Even with such limitations, there was signal of improved risk model predictive ability in women age 40 to 49 compared with older women. Additionally, cholesterol values for some studies were not available in all subjects, thus values were predicted and not directly measured. Thus, further study is warranted to assess, in a rigorous fashion with comprehensive CVD endpoints, validated outcomes, and consistent variable measurements, whether APO incorporation truly leads to improved risk prediction, especially in young women in whom risk modification may mitigate future CVD risk.

## 3 ANTEPARTUM CVD PREVENTION FOR WOMEN AT INCREASED RISK OF APOs

### 3.1 The role of aspirin therapy

Data have emerged on the utility of aspirin therapy in early pregnancy for women at high‐risk for either preeclampsia or preterm birth. A systematic review and meta‐analysis by Roberge et al^21^ concluded that aspirin administration starting at or <16 weeks gestation reduces the risk of preterm preeclampsia up to 70%.^21^ A recent study in 11 000 women also found aspirin was effective in reducing risk of preterm birth in nulliparous women with history of singleton pregnancy.^22^ The American College of Obstetricians and Gynecologists (ACOG) recommends low‐dose aspirin 81 mg/d prophylaxis in women at high risk for preeclampsia, initiated between 12 and 28 weeks gestation (optimally before 16 weeks) and continued daily up to delivery. The mechanism of action behind aspirin use may be related to aiding healthy placentation; uterine spiral artery remodeling is completed by 16 to 18 weeks.^23^

Educating women's health providers on utilizing preeclampsia prediction algorithms and empirically treating high‐risk patients with timely low‐dose aspirin therapy is imperative. Improving risk prediction models in preeclampsia, in particular for nulliparous women, is important for early initiation of prevention strategies, although whether prevention of preeclampsia reduces future CVD risk remains to be determined. Whether a woman with history of preeclampsia benefits from longer‐term strategies such as aspirin is also unknown. As personalized medicine continues to emerge, the cardiovascular community can play an active, preventive role in research, and discovering new treatments for these high‐risk APO women at risk of CVD later in life.

### 3.2 Antepartum blood pressure management

HDPs of pregnancy are the leading cause of maternal morbidity and mortality, contributing to 14% of maternal deaths world‐wide.^24^ The ACOG^25^ identifies the importance of identification and treatment of acute onset, severe hypertension in pregnant or postpartum women, emphasizing, “the goal is not to normalize blood pressure, but to achieve a range of SBP 140–150/ DBP 90–100 mm Hg in order to prevent repeated, prolonged exposure to severe systolic hypertension.”^25^ More commonly seen is gestational hypertension defined as onset of hypertension (SBP ≥ 140 mm Hg and/or DBP ≥ 90 mm Hg) after 20 weeks of gestation without proteinuria or absence of new signs of end‐organ dysfunction. The diagnosis evolves into preeclampsia if new‐onset proteinuria or end‐organ dysfunction accompany elevated blood pressure.

The ACOG Practice Bulletin on Chronic Hypertension in Pregnancy^26^ acknowledged its conflict with the 2017 ACC/AHA Blood Pressure Guideline recommendations, as the hypertension diagnosis threshold for nonpregnant women is lower compared with ACOG's criteria. The ACC/AHA guidelines recommend that nonpregnant patients with stage 1 hypertension (SBP 130‐139 mm Hg and/or DBP 80‐89 mm Hg) with CVD risk factors should begin treatment. While ACOG states that it is reasonable to continue to manage a chronic hypertensive patient in pregnancy similarly to the ACC/AHA guidelines for chronic hypertension, there is no recommendation for management of a gestational hypertensive woman within the stage 1 range. A secondary analysis of a low‐dose aspirin trial for preeclampsia prevention found that aspirin did not appear to lower the risk of preeclampsia in the group redefined as stage 1 hypertensive.^27^ Application of the ACC/AHA blood pressure guidelines to pregnant women thus needs to be an active area of research. Without contemporary data and randomized controlled trials demonstrating improved outcomes with lower hypertension thresholds, it is difficult to translate current CVD guidelines to an antepartum population. For example, the Control of Hypertension in Pregnancy Study (CHIPS) randomized trial of “less‐tight” control (target DBP 100 mm Hg) or “tight” control (target DBP 85 mm Hg) demonstrated no significant differences in the risk of pregnancy loss, high‐level neonatal care, or overall maternal complications, although less‐tight control was associated with higher frequency of severe maternal hypertension.^28^ Research is needed to determine whether lower blood pressure thresholds and treatment targets in pregnancy improve long‐term CVD outcomes.

### 3.3 Cholesterol and APOs

Cholesterol levels normally increase in pregnancy, however, women with preeclampsia have higher serum triglyceride (TG) and lower high density lipoprotein (HDL) levels compared with those without preeclampsia.^29^ During pregnancy, TG levels rise during each trimester; however, women with hypertriglyceridemia (TG ≥ 500 mg/dl [5.6 mmol/L]) prepregnancy may develop severe third‐trimester hypertriglyceridemia, placing them at increased risk for pancreatitis.^30^ Pregnancy also affects low‐density lipoprotein (LDL) cholesterol levels, which can exceed 190 mg/dl (4.9 mmol/L) in the third trimester.^31^ This can raise questions of heterozygous familial hypercholesterolemia (FH) if pre‐pregnancy LDL values are not known. If a woman has a first‐degree relative with premature CVD, screening for FH should be completed in the postpartum setting (within the first 12 weeks).^32^ Other APOs, such as spontaneous preterm birth, also demonstrate lower HDL and higher TG levels throughout a woman's life course.^33^, ^34^ Optimally managing healthy lifestyle habits should be discussed first in women with dyslipidemias.^5^ If a woman on HMG‐CoA reductase inhibitor (statin) therapy is considering pregnancy, she should stop her statin 1 to 2 months prior to contraception cessation. If the pregnancy is unplanned, she should stop it as soon as she is aware.^5^

Statins are currently contraindicated during pregnancy.^35^ However, statins are known to have pleiotropic effects, which may diminish inflammation and oxidative stress, increase angiogenesis, inhibit the coagulation cascade, and protect the endothelium. Data suggests that a particular statin, pravastatin, has a relatively safer pharmacologic profile in pregnancy compared to other statins in animal models.^36^ Human clinical trials are currently in progress to determine whether a hydrophilic statin may be used to prevent preeclampsia in high‐risk women.^37^, ^38^

### 3.4 Gestational diabetes management

Gestational diabetes is associated with a 2‐4‐fold increased risk of preeclampsia^39^ and a 2‐fold increased future CVD risk, with CVD events occurring 7 years earlier in women with gestational diabetes compared with healthy women.^40^ As gestational diabetes and preeclampsia share risk factors, including poor glycemic control and prepregnancy obesity, appropriate management of these risk factors, including prepregnancy weight loss, nutritional therapy, moderate exercise, glucose monitoring, and insulin therapy (if target glucose levels are not achieved with diet alone) are recommended by the American Diabetes Association (ADA) and ACOG.^41^, ^42^ Insulin is the preferred medication for treating hyperglycemia as it does not cross the placenta to a measurable extent. For those with preexisting diabetes, the ADA recommends preconception counseling to address glycemic management goals of HgbA1c <6.5% to reduce the risk of APOs and congenital abnormalities. Since pregnancy HgbA1c values are slightly lower due to increased red blood cell turnover, the ADA recommends an ideal pregnancy A1c target to be <6%, although this may be relaxed to <7% to prevent hypoglycemia. Studies are needed to establish whether intensive glucose control during pregnancy reduces long‐term CVD risk.

## 4 BEST PRACTICES FOR CVD PREVENTION IN THE POSTPARTUM SETTING

For most women, pregnancy and the postpartum setting provide a window of opportunity for CVD risk screening. Approximately 20% of women will have one or more APOs that are associated with CVD risk factors and future CVD.^43^ The presence of APOs is particularly important in minorities such as African American women who are at highest risk for adverse maternal outcomes. Therefore, implementing postpartum screening and intervention strategies could improve long‐term cardiovascular health and future pregnancy outcomes particularly in underserved populations. Promotion of breast feeding, which has been associated with reduced risk of diabetes, is also important for both maternal and infant outcomes.^44^ After pregnancy and throughout the life course of every woman, a comprehensive pregnancy history should be obtained, and risk factors and risk‐enhancing factors (premature menopause with onset age <40 years, history of preeclampsia) should be identified.

Unfortunately, many healthcare providers are not aware of the association between pregnancy complications, the presence of CVD risk factors, and the development of future CVD.^45^ Better knowledge translation is required to facilitate timely access to care for health preservation and disease prevention. The complications of pregnancy that are associated with future CVD usually will be managed by obstetricians and/or maternal‐fetal medicine specialists, who should be planning or consulting for longer‐term follow‐up at the time these patients are identified, which is usually late in pregnancy or at delivery. Inclusion of this consultative process in standard postpartum order sets will facilitate referral and ensure patients with complications are not missed. Specialized programs exist throughout the United States and Canada. One such program, *The Maternal Health Clinic* ^43^ in Kingston, Canada, has created a “Maternal Health Clinic Handbook” to help healthcare providers initiate and run a clinic and include examples of data‐base forms (https://www.themothersprogram.ca/for-care-providers/the-postpartum-maternal-health-clinic-handbookc). It is important to discuss the rationale for the referral with the woman prior to discharge from the hospital to increase the chance that she will attend for follow‐up and screening.

It is recommended that these women initiate screening within 3 to 6 months postpartum,^46^ in part to allow time for improvement in physical and/or biochemical risks prior to a future pregnancy. In women with HDP, closer follow‐up in the postpartum period is indicated as the maternal mortality rate in a state representative sample is highest during the first 6 weeks after delivery.^47^ Recommendations for comprehensive screening are presented in the Table 1. Blood pressure should be managed as per the ACC/AHA guidelines.^48^ Abnormal lipid profiles should be repeated postpartum following a period of lifestyle modification (ie, 6‐12 months), and if they are persistently elevated the provider should initiate statin therapy.^5^

**TABLE 1:** Recommendations for assessment of adverse pregnancy outcomes and screening for cardiac risk factors

*Pregnancy history* Hypertensive disorders of pregnancy Gestational hypertension Preeclampsia Eclampsia Chronic hypertension Gestational diabetes Preterm birth (<37 weeks) Intrauterine growth restriction/low birth weight/small for gestational age *Medical history* Tobacco use Physical activity Breast feeding history Hypertension Diabetes Family history of cardiovascular disease *Physical Examination* Blood pressure and heart rate Body mass index and waist circumference *Laboratory evaluation (yearly)* Lipid profile Fasting glucose/HgA1c Urine protein evaluation

*Source:* Modified from 2019 ACOG Practice Bulletin—Clinical Management Guidelines for Obstetrician‐Gynecologists.

Excess weight gain during pregnancy and postpartum weight retention and/or weight gain are all associated with future obesity.^49^ Body weight is a strong independent risk factor in women for the development of CVD, and each body mass index unit increase amounts to a 10% increase in risk for CVD.^50^ Behavioral‐based prevention strategies for facilitation of weight loss postpartum through dietary and lifestyle intervention, encouraging regular physical activity, and maintaining a healthy BMI, are appropriate strategies to prevent maternal obesity, thereby reducing CVD risk. Pregnancy and the postpartum period are considered a “teachable moment” for weight control and obesity prevention.^51^

While a number of specialized clinics have been initiated across the United States and Canada, most women will not be able to be seen in these clinics. Therefore, all providers who take care of postpartum women, (ie, obstetricians/maternal‐fetal medicine, internists, cardiologists, family physicians, and nurse practitioners) should initiate screening and recommend lifestyle interventions. Allied healthcare providers who can assist with behavioral and lifestyle modification (eg, dieticians, social workers, clinical psychologists, physical therapists, and personal trainers) should also be involved. Practitioners should also be particularly vigilant in screening for APOs and implementing lifestyle changes in minority women. In such populations, access to care may be limited and further resources should be dedicated to focusing on these underserved populations of women with APOs and who often exhibit significant baseline cardiac risk factors and are at high risk of CVD.

In addition the immediate postpartum period, awareness of APO and CVD risk association is crucial to risk reduction throughout a woman's life course. Although the postpartum period is an ideal time to discuss with a patient their particular APO‐CVD risk association, a woman's CVD risk continues to increase with age particularly as they approach menopause. As such, screening for APOs at any period even remotely from delivery is a key to reduce CVD risk and implementing lifestyle and screening measures as described here.

## 5 FUTURE DIRECTIONS

While the association between APOs and long‐term maternal CVD is well‐established, what to do with this information is less so. Recommendations for follow‐up and testing are largely based on benefits obtained in older women. Future studies should focus on the efficacy and effectiveneness of various testing and management approaches. Decision and cost‐effectiveness analyses would also inform which approaches are most optimal from public health and health policy perspectives.

The impact of the recent guidelines for diagnosis and treatment of hypertension on the association of APOs with long‐term maternal CVD needs to be evaluated. More women, particularly younger women, will start pregnancy with a diagnosis of pregestational hypertension, or may already be taking antihypertensive medications. Pregnancy outcomes and diagnosis of preeclampsia will certainly be affected by these changes. As most of the current evidence on long‐term outcomes of women with APOs is based on the traditional definition of hypertension, we expect that more women would be diagnosed with hypertension and receive treatment than what is currently estimated.

Additional research is also required to improve our understanding of the association between APOs and long‐term maternal health. Mechanistic studies are needed to determine whether this association is causative. If APOs are partially or wholly causative of long‐term CVD, then approaches to prevent these APOs become of high public health interest.

Loss of healthcare coverage after the 6 weeks postpartum period is a major limitation to continuing the care of women with APOs. Training of clinicians who can manage these patients should also include awareness of such issues to facilitate risk modification in such patients. Recent guidelines from major cardiovascular and obstetric societies have also stressed the importance of APOs and CVD risk and further efforts to promote interdisciplinary collaboration between cardiologists, obstetricians/maternal‐fetal medicine specialists and primary care providers are key to improving CVD outcomes in women.^52^

## DISCLOSURE OF INTERESTS

Dr Minissian reports consulting with Amgen, Medical Advisory Board; honorarium NACCME, LLC Co‐Chair for CME; Vox Media; Medtelligence; Minneapolis Heart Institute; Primed; Good Samaritan Hospital, Los Angeles, CA; Cardiometabolic Health Congress; American Heart Association; National Lipid Association; Preventive Cardiovascular Nurses Association; American College of Cardiology. All other authors have no disclosures to report. The content is solely the responsibility of the authors and does not necessarily represent the official views of the National Institutes of Health.
